# Reduction of Dislocation of the Femur

**Published:** 1852-01

**Authors:** 


*Reduction of Dislocation of the Femur.*—In a Report of the Surgical wards of the *Pennsylvania Hospital*, contained in the *Medical Examiner* for Dec., 1851, is included a case of dislocation of the femur. In a foot note it is stated, "Reduced in ten minutes, under the influence of ether, *after three previous unsuccessful attempts without it, one of which lasted three hours.*" We have italicised a part of the quotation. Was the method proposed by Dr. Reid tried in this case, or has an account of this method not yet reached Philadelphia?

